# Case Report: Management of recurrent UTI with tigecycline in a kidney transplant recipient

**DOI:** 10.3389/frtra.2024.1496702

**Published:** 2025-02-18

**Authors:** Supreeta R. Shettar, Mahadevaiah Neelambike Sumana, Manjunath S. Shetty, Yogeesh D. Maheshwarappa, Raghukanth G. Reddy, Asha Srinivasan, Vamshi Dharan P, Gautam Kalyatanda, Shruthi Shree S C

**Affiliations:** ^1^ JSS Medical College and Hospital, JSS Academy of Higher Education and Research, Mysuru, India; ^2^ Division of Nanoscience and Technology, School of Life Sciences, JSS Academy of Higher Education and Research, Mysuru, India; ^3^ Division of Infectious Disease and Global Medicine, University of Florida, Gainesville, FL, United States

**Keywords:** multi-drug resistant *Klebsiella pneumoniae*, multidrug-resistant organisms, urinary tract infection, kidney transplantation, immunocompromised conditions

## Abstract

This case report describes a 37-year-old man who underwent renal transplantation and subsequently developed complicated recurrent urinary tract infections (UTIs) caused by multidrug-resistant (MDR) *Klebsiella pneumoniae*. Despite initial treatment with fosfomycin and meropenem, the patient experienced persistent UTIs, leading to multiple hospitalizations. The management of these recurrent infections eventually required the use of tigecycline. Although tigecycline is not typically considered a urinary antibiotic owing to its limited urinary excretion, it was successfully employed in this case to manage the recurrent infections. The patient was treated with tigecycline for several episodes of UTI, which provided a crucial therapeutic option in the context of antibiotic resistance. This case underscores the challenges of managing recurrent MDR UTIs in immunocompromised patients and highlights tigecycline as an effective treatment strategy when standard therapies fail.

## Introduction

Urinary tract infections (UTIs) are the most common complication among kidney transplant (KTx) recipients, potentially reducing the transplant kidney's long-term function and increasing the risk of mortality for the recipient (1). Any symptomatic UTI in a transplant patient is considered complicated, regardless of whether it affects the lower or upper urinary tract, due to the increased susceptibility to infections and treatment concerns associated with immunosuppression (2). Although UTIs can arise at any time after transplantation, they are most common during the first year, particularly within the first 3–6 months (3). The principal causes of UTIs in KTx recipients are similar to those observed in the general population with complicated UTIs (4). Gram-negative bacteria cause up to 90% of UTIs after kidney transplantation, with *Klebsiella pneumoniae* emerging as a prominent causative agent globally, particularly in cases of recurrent infections involving multidrug-resistant (MDR) strains, in KTx recipients with urinary flow abnormalities, such as ureteral stenosis, or underlying urological issues, such as neurogenic bladder or chronic vesicoureteral reflux (5).

*K. pneumoniae* is a gram-negative bacterium from the Enterobacteriaceae family that is encapsulated and non-motile (6). It is part of the normal flora and colonizes numerous human mucosal surfaces, including the distal urethra, upper respiratory tract, and gastrointestinal tract (6). It is regarded as an opportunistic pathogen that is frequently implicated in severe hospital-acquired infections, especially among immunocompromised patients and those with significant comorbidities. *K. pneumoniae* causes a wide range of infections, including pneumonia, UTIs, bacteremia, and liver abscess (7).

The emergence of multidrug-resistant *K. pneumoniae* is accelerated by antibiotic selection pressure. Resistance, particularly to carbapenems due to carbapenamase production, has increased over the last decade, posing a significant threat to healthcare systems worldwide (8). The inclusion of extended-spectrum β-lactamase (ESBL) genes on large plasmids, coupled with genes for resistance to other antimicrobial drugs, facilitates this resistance, resulting in a multidrug-resistant phenotype. This mechanism is frequently associated with ESBL-producing Enterobacteriaceae like *K. pneumoniae* (9). ESBL-producing organisms may develop resistance to other antibiotics routinely used for UTIs, such as fluoroquinolones, cotrimoxazole, or aminoglycosides, which are challenging to manage (1). Consequently, careful selection of empirical antibiotic therapy for UTIs is critical, and it should be guided by risk factors associated with infections. Here, we describe a case of multidrug-resistant *K. pneumoniae* isolated from a renal transplant recipient with recurrent UTI who was treated with tigecycline, although tigecycline is not a good urinary antibiotic. Written consent was obtained from the patient to present this case.

## Case presentation

A 37-year-old male patient with a known history of hypertension, obesity, and obstructive sleep apnea who was diagnosed with end-stage kidney disease underwent renal transplantation from a live-related donor (donor-mother). A double J (DJ) stent was deployed during surgery. After transplantation, the patient was treated with triple immunosuppressants, namely, prednisolone, mycophenolate, and tacrolimus. He had no postoperative complications. The DJ stent was removed on postoperative day 9. The patient had good allograft function with a serum creatinine of 1.1 mg/dl at discharge. On day 14 after the transplant, the patient visited the outpatient department for follow-up. Although asymptomatic, his urine culture showed the growth of multidrug-resistant *K. pneumoniae*, which was treated with 3 g of fosfomycin once in 3 days given during his recent renal transplant according to the culture sensitivity report as per standard guidelines (10–12). One week later (post-transplant day 21), repeat urine culture still showed multidrug-resistant *K. pneumoniae*, and treatment with fosfomycin was continued for another three doses as per standard guidelines. Two months after the previous treatment with fosfomycin, the urine culture yielded no growth (13, 14). The timeline of treatment is given in Table 1 and the antibiotic susceptibility report (AST) report is given in Table 2.

**Table 1:** Episodes of care.

Treatment timeline	Treatment given
1. Post-transplantation	The patient was treated with triple immunosuppressants, namely, prednisolone, mycophenolate, and tacrolimus
2. 14th post-transplant day	The patient was treated with 3 g of fosfomycin once in 3 days
Urine culture yielded MDR *K. pneumoniae* (AST report in Table 2)	
The patient was improved	
3. 21st post-transplant day	The patient was treated with another three doses of fosfomycin (3 g)
Repeat urine culture yielded MDR *K. pneumoniae* (AST report in Table 2)	
The patient was improved repeat urine culture showed no growth	
4. 4 months post-transplant	Cefoparazone sulbactam 1.5 gm 12th hourly for 7 days
Urine culture yielded *E. coli* (AST report in Table 3)	
The patient was improved symptomatically and discharged from the hospital	
5. 5 months post-transplant	IV fluconazole (150 mg) for 2 weeks for esophageal candidiasis, and meropenem 1 g 8th hourly over a 3-h infusion for 2 weeks to treat the MDR *K. pneumoniae*
The patient was diagnosed with esophageal candidiasis along with UTI caused by MDR K. *pneumoniae* (AST report in Table 2)	
Patient responded to treatment and discharged consequently	
6. 6 months post-transplant	Meropenem 1 g 8th hourly over a 3 h infusion for 14 days and oral levofloxacin 500 mg OD for 5 days because of the immunosuppression. The patient was also treated with oseltamivir because of the ongoing H1N1 pandemic
Sputum culture insignificant bacterial growth and ongoing H1N1 pandemic	
The patient was improved and discharged from the hospital	
7. 9 months post-transplant	Tigecycline 100 mg stat dose followed by 50 mg BD for 3 days for conservative management (in view of previous infection with MDR GNB)
For left epididymal orchitis	
8. After left orchidectomy	IV imipenem + cilastatin 500 mg BD for 10 days
Urine culture yielded MDR *K. pneumoniae* (AST report in Table 4)	
The patient was improved symptomatically and discharged from the hospital	
9. 1 month post-orchidectomy/10 months post-transplant	IV tigecycline 50 mg BD for 3 days
Urine culture yielded MDR *K. pneumoniae* (AST report in Table 4)	
The patient was improved	
10. 11 months post-transplant	IV tigecycline 100 mg stat dose followed by 50 mg BD for 10 days
After TURP urine culture yielded MDR *K. pneumoniae* (AST report in Table 4)	
Symptomatically the patient began to improve and there was no further recurrence of UTI to date.	

MDR, multidrug-resistant; IV, intravenous; TURP, transurethral resection of the prostate; AST, antibiotic susceptibility report; GNB, gram negative bacilli.

**Table 2:** Antimicrobial susceptibility pattern of MDR *K. pneumoniae* (before left orchidectomy).

Antimicrobial agent	14th post-transplant day		21st post-transplant day		5 months post-transplant	
	MIC	Interpretation	MIC	Interpretation	MIC	Interpretation
Amoxicillin and clavulanic acid	≥32	Resistant	≥32	Resistant	≥32	Resistant
Piperacillin-tazobactam	≥128	Resistant	≥128	Resistant	≥128	Resistant
Cefuroxime	≥64	Resistant	≥64	Resistant	≥64	Resistant
Cefuroxime axetil	≥64	Resistant	≥64	Resistant	≥64	Resistant
Ceftriaxone	≥64	Resistant	≥64	Resistant	≥64	Resistant
Cefaperazone-sulbactam	≥64	Resistant	≥64	Resistant	≥64	Resistant
Cefepime	≥32	Resistant	≥32	Resistant	≥32	Resistant
Ertapenem	≥8	Resistant	≥8	Resistant	≥8	Resistant
Imipenem	8	Resistant	8	Resistant	8	Resistant
Meropenem	≥64	Resistant	≥64	Resistant	≥64	Resistant
Amikacin	32	Resistant	32	Resistant	32	Resistant
Gentamicin	≥16	Resistant	≥16	Resistant	≥16	Resistant
Ciprofloxacin	≥4	Resistant	≥4	Resistant	≥4	Resistant
Tigecycline	2	Sensitive	≥8	Resistant	≥8	Resistant
Fosfomycin	—	Sensitive	—	Sensitive	—	Resistant
Trimethoprim-sulfamethoxazole	≥320	Resistant	≥320	Resistant	≥320	Resistant
Colistin	≤0.5	Intermediate	≤0.5	Intermediate	≤0.5	Intermediate
Nitrofurantoin	—	Resistant	—	Resistant	—	Resistant

MIC, minimum inhibitory concentration.

Approximately 4 months after the transplant, the patient developed bilateral lower limb swelling. On evaluation, the patient was found to have proteinuria and erythrocytosis and a transplant kidney biopsy was performed that showed focal segmental glomerulosclerosis (FSGS) with interstitial fibrosis and tubular atrophy (IFTA)-20%, for which he was treated conservatively. In addition, because of his urine showing pus cells, a repeat urine culture was performed that showed the growth of *Escherichia coli* and the patient was treated with cefoparazone/sulbactam 1.5 g every 12 h for 7 days. The AST report is given in Table 3.

**Table 3:** Antimicrobial susceptibility pattern of *E. coli* isolated in the fourth month after transplantation.

Antimicrobial agent	MIC	Interpretation
Amoxicillin and clavulanic acid	≥32	Resistant
Piperacillin-tazobactam	≥128	Resistant
Cefuroxime	≥64	Resistant
Cefuroxime axetil	≥64	Resistant
Ceftriaxone	≥64	Resistant
Cefaperazone-sulbactam	32	Sensitive
Cefepime	≥32	Resistant
Ertapenem	0.25	Sensitive
Imipenem	≥0.25	Sensitive
Meropenem	≥0.25	Sensitive
Amikacin	4	Sensitive
Gentamicin	≥16	Resistant
Ciprofloxacin	≥4	Resistant
Tigecycline	2	Sensitive
Fosfomycin	—	Sensitive
Trimethoprim-sulfamethoxazole	≥320	Resistant
Colistin	≤0.5	Intermediate

Five months after the transplant, the patient presented with generalized weakness, sweating, and joint pain for 1 week. On evaluation, he was found to have uncontrolled sugar levels leading to esophageal candidiasis along with a UTI caused by multidrug-resistant *K. pneumoniae*. He was treated with intravenous (IV) fluconazole (150 mg) for 2 weeks for esophageal candidiasis, and meropenem 1 g every 8 h over a 3-h infusion for 2 weeks to treat the MDR *K. pneumoniae* causing the UTI. An insulin infusion was started for uncontrolled diabetes. The AST report is given in Table 2.

Six months after the transplant, the patient, with a history of new-onset diabetes after transplant (NODAT), presented with cytomegalovirus (CMV) syndrome. He presented with low-grade fever, cough with expectoration, generalized weakness fatigue, rhinorrhea, and cold. On examination, the patient was febrile and had bilateral basilar crepitations and diffuse rhonchi on auscultation. A chest radiograph showed non-homogeneous opacities in the right lower zone for which he was admitted. In the pulmonologist's opinion, he underwent high-resolution computed tomography (HRCT) of the thorax, which showed randomly distributed centrilobular nodules in a tree bud configuration with adjacent ground-glass opacities and mediastinal lymphadenopathy in favor of active infective etiology. The sputum sample sent for Gram staining showed no significant findings, and the Potassium hydroxide (KOH) test result was negative. H1N1 RTPCR and COVID RTPCR results were also negative. Sputum culture also revealed no significant pathogenic bacterial growth. The patient was treated with antibiotics meropenem 1 g ever 8 h over a 3-h infusion for 14 days and oral levofloxacin 500 mg once daily (OD) for 5 days because of the immunosuppression. He was also treated with oseltamivir due to the ongoing H1N1 pandemic and was later discharged.

Nine months after the transplant, the patient presented with complaints of swelling in the left scrotal region with severe pain. He was diagnosed with left epididymal orchitis. A urology opinion was sought, which was managed conservatively with antibiotics and pain medications. During the hospital stay, he was treated with IV antibiotic tigecycline 100 mg stat dose followed by 50 mg twice daily (BD) for 3 days for conservative management. However, the patient continued to have severe pain in the left testis and underwent a left orchidectomy. Tigecycline was preferred over other antibiotics given the possibility of infection with MDR as he was an immunosuppressed patient with multiple antibiotics for managing previous infections. The tigecycline therapy was initiated based on the Indian Council of Medical Research (ICMR) treatment guidelines, which suggest the use of antibiotics such as ceftriaxone, ofloxacin, levofloxacin, and doxycycline for managing infections. Tigecycline, similar to doxycycline as a glycylcycline, was chosen based on studies demonstrating its successful use in treating complicated UTIs (13, 15, 16).

A urine culture showed the growth of MDR *K. pneumoniae*. As per the sensitivity report, the patient was started on IV imipenem + cilastatin 500 mg BD for 10 days and was discharged when he was symptomatically better. One month after the orchidectomy, he presented with fever and chills. On investigation he was found to have a UTI, and urine culture showed the growth of multidrug-resistant *K. pneumoniae*. The AST report is shown in Table 4. The patient was treated with IV tigecycline 50 mg BD for 3 days. The patient underwent a diagnostic cystoscopy because of post-void residual (PVR) urine, which showed benign prostatic hyperplasia (BPH)—grade I lateral lobes with obstructed bladder and urodynamics showed bladder outlet obstruction.

**Table 4:** Antimicrobial susceptibility pattern of MDR *K. pneumoniae* (after left orchidectomy).

Antimicrobial agent	10 months post-transplant/1 month post-left orchidectomy		11 months post-transplant	
	MIC	Interpretation	MIC	Interpretation
Amoxicillin and clavulanic acid	≥32	Resistant	≥32	Resistant
Piperacillin-tazobactam	≥128	Resistant	≥128	Resistant
Cefuroxime	≥64	Resistant	≥64	Resistant
Cefuroxime axetil	≥64	Resistant	≥64	Resistant
Ceftriaxone	≥64	Resistant	≥64	Resistant
Cefaperazone-sulbactam	≥64	Resistant	≥64	Resistant
Cefepime	≥32	Resistant	≥32	Resistant
Ertapenem	≥8	Resistant	≥8	Resistant
Imipenem	4	Sensitive	≥8	Resistant
Meropenem	2	Sensitive	≥16	Resistant
Amikacin	32	Resistant	32	Resistant
Gentamicin	≥16	Resistant	≥16	Resistant
Ciprofloxacin	≥4	Resistant	≥4	Resistant
Tigecycline	2	Sensitive	2	Sensitive
Fosfomycin	—	Resistant	—	Sensitive
Trimethoprim-sulfamethoxazole	≥320	Resistant	≥320	Resistant
Colistin	≤0.5	Intermediate	≤0.5	Intermediate

At 11  months after transplantation, the patient presented with recurrent fever and burning micturition. Because of the bladder outlet obstruction with significant post-void residual urine, he underwent a bladder neck incision with a partial transurethral resection of the prostate (TURP); repeat urine culture showed plenty of inflammatory cells in urine microscopy and the growth of multidrug-resistant *K. pneumoniae*, which was susceptible only to tigecycline, as shown in Table 4. In addition, the organism was resistant to ceftazidime-avibactam and aztreonam, and negative for synergy between ceftazidime-avibactam and aztreonam. The testing procedure was carried out according to the Clinical and Laboratory Standards Institute (CLSI) guidelines (17). Hence, he was treated with IV tigecycline 100 mg stat dose followed by 50 mg BD for 10 days. Symptomatically, the patient began to improve and there was no further recurrence of the UTI to date.

During the therapeutic process, the patient underwent a personalized regimen addressing individual concerns, such as incorporating antimicrobial therapies (fosfomycin, meropenem, levofloxacin, fluconazole, imipenem + cilastatin, and tigecycline), surgical interventions (orchidectomy, bladder neck incision with partial transurethral resection of the prostate), and immunosuppressive protocols (prednisolone, mycophenolate, and tacrolimus). Despite encountering obstacles, the patient exhibited stability after the surgeries and interventions.

## Discussion

UTIs caused by multidrug-resistant Enterobacteriaceae, such as *K. pneumoniae*, pose a substantial clinical issue (18). Patients infected with MDR pathogens frequently have more severe infections, exacerbating the limited antibiotic alternatives available for treating multidrug-resistant pathogens like *K. pneumoniae* that cause UTIs (19). The overuse of β-lactam antibiotics has caused changes in *K. pneumoniae* strains and increased the synthesis of β-lactamases, leading to multidrug resistance, to broad-spectrum cephalosporins and carbapenems (19). Aminoglycosides and polymixins are viable alternatives for treating infections resistant to β-lactams and fluoroquinolones. Still, these options are ruled out in patients with renal impairments, especially in transplant patients due to the nephrotoxicity of these agents (9). Tigecycline is a tetracycline-class antibacterial agent designed to combat polymicrobial MDR infections. Marketed as GAR-936 or Tygacil, it is the first and only member of the glycylcycline class of semi-synthetic agents and is administered parenterally. Its primary mechanism of action involves the inhibition of bacterial protein synthesis. Tigecycline binds reversibly to the helical region (H34) on the 30S subunit of bacterial ribosomes, interfering with the elongation of the peptide chain. This prevents the incorporation of amino acid residues, halting peptide formation and bacterial growth (20, 21).

Tigecycline, a tetracycline-based parenteral antibiotic with broad-spectrum efficacy against both Gram-positive and Gram-negative bacteria, is one last-resort antibiotic that can be used to treat multidrug-resistant infections. Although tigecycline is not traditionally regarded as an ideal choice for UTIs due to its low serum concentration and limited urinary excretion (approximately 33% of the administered dose is excreted unchanged in the urine), recent studies have reported successful outcomes in treating multidrug-resistant complicated UTIs. These cases suggest that tigecycline can serve as a last-resort drug, especially in patients with MDR infections in cases of renal disease, offering a less toxic alternative when other options fail (15, 22–24). Although it is primarily prescribed for abdominal and skin/soft-tissue infections, its efficacy in treating UTIs is questionable (25). Tigecycline has been successful in some UTI cases, with greater doses being more effective against multidrug-resistant organisms due to its pharmacokinetic profile and increasing urine concentration (26). Furthermore, tigecycline is more resistant to common bacterial resistance mechanisms like enzymatic inactivation, target site alterations, and efflux pump activation (27).

In the present case, a renal transplant recipient with recurrent UTI infection caused by multidrug-resistant *K. pneumoniae* was treated with tigecycline and achieved favorable outcomes. The clinical guidelines (according to ICMR 2022 and Infectious Disease Society of America (IDSA) 2023) indicate the use of antibiotics like meropenem, imipenem + cilastatin, and aminoglycosides as an alternative option for the treatment of complicated UTIs due to MDR infections. However, in this case, the patient did not respond to the antibiotic treatment of meropenem and imipenem + cilastatin. Aminoglycosides cannot be used due to nephrotoxicity. Hence, treatment with tigecycline was initiated even though it is not recommended in the clinical guidelines. However, according to the existing literature, tigecycline is a viable second-line option to treat UTIs caused by MDR organisms. For the treatment of UTIs with tigecycline, the dosage of 100 mg followed by 50 mg twice daily appeared to be associated with positive clinical outcomes; therefore, the same dosage was initiated for the patient (28).

In a systematic review conducted by Liu et al., comprising 31 different complicated UTI cases, which included comorbid conditions like transplantation, diabetes mellitus, catheter-associated UTI, end-stage renal disease, prostatitis, surgery, and pulmonary disease, the majority of the cases involved transplantation. Among the 31 cases, 14 were infected with multidrug-resistant *K. pneumoniae* and were treated with tigecycline. In 9 out of 14 cases, treatment with tigecycline was effective; however, the outcomes were not documented in the remaining five cases due to insufficient data on the specific microbiological status (20). Similarly, Wu et al. conducted a comprehensive study that included discussions on several cases of recurrent UTI caused by multidrug pathogens like *E. coli*, *Acinetobacter baumannii*, and *K. pneumoniae*. In all of the cases, treatment with tigecycline was initiated either because of MDR pathogens or due to inadequate response to previous antibiotic therapies. In the majority of cases, tigecycline was effective in treating complicated or recurrent UTIs caused by multidrug-resistant pathogens (14). Reema et al. (16) conducted a systematic review that included 27 cases of complicated UTIs, all of which were treated with tigecycline. Of the 27 cases, 11 were caused by multidrug-resistant *K. pneumoniae*, while the remaining cases were caused by other Gram-negative bacteria such as *E. coli* and *A. baumannii*. The average duration of tigecycline treatment was 13 days. In 24 cases, the clinical result was favorable, with only 4 individuals experiencing UTI recurrence (16).

The current case contributes to the existing literature by highlighting the effectiveness of tigecycline in treating patients with complicated UTIs caused by MDR bacteria, in which tigecycline is the sole susceptible antibiotic. The success of tigecycline treatment in complicated UTIs can be attributed to several factors: its status as a relatively newer glycylcycline; the sensitivity of multidrug-resistant Gram-negative bacteria to tigecycline even at lower concentrations; and its resistance to common bacterial resistance mechanisms, including enzyme inactivation, target site alterations, and efflux pump activation (27).

## Strengths of the study

1. Although the patient had financial constraints and the newer β-lactams were not available for the treatment of MDR infections according to the latest ICMR and IDSA guidelines, the patient and the whole team of doctors did not give up. Alternative, available options were explored and the patient was treated successfully.
2. This report provides valuable insights into the use of tigecycline as a last resort for the treatment of complicated UTIs caused by MDR organisms, specifically MDR *K. pneumoniae*. It highlights the potential efficacy of tigecycline, particularly in cases where conventional therapies have failed.
3. Managing infections in immunocompromised patients, such as renal transplant recipients, presents unique challenges. This report underscores the complexity of treating recurrent MDR infections in transplant patients, highlighting tigecycline's potential role in such difficult clinical scenarios.
4. This case report also demonstrates the importance of individualized treatment strategies, especially when managing complex cases involving antibiotic resistance, immunosuppression, and recurrent infections. It also illustrates the need for adaptive therapeutic approaches when standard treatments fail.

## Limitation of the study

1. Due to the non-availability of newer β-lactams like ceftolozane-tazobactam, imipenem-cilastatin-relebactam, meropenem-vaborbactam, cefepime-zidebactam in India, the sensitivity of organism isolated could not be tested for these drugs and hence could not be used on this patient for treatment.
2. Due to the patient’s financial constraints, tigecycline was administered for only 3 days during the initial phases of treatment, which likely contributed to recurrent infections. However, during the most recent episode, tigecycline was used for 10 days, resulting in the resolution of the infection.
3. This case report details the successful use of tigecycline in a single case, which may not be generalizable to all renal transplant recipients with multidrug-resistant UTIs. Further studies are needed to confirm the efficacy and safety of tigecycline in similar patient populations. In addition, while this case adds to the existing literature on the use of tigecycline for MDR UTIs, high-quality randomized controlled trials are required to determine whether tigecycline can be systematically recommended in such cases, given the current lack of robust clinical evidence for its efficacy in complicated UTIs in low- and middle-income countries.
4. Due to financial constraints, molecular testing could not be performed on the isolated MDR *K. pneumoniae* treated with tigecycline.

## Conclusion

Given the rise in antibiotic resistance, the use of antibiotics like tigecycline, which has improved activity against multidrug-resistant pathogens, may see increased use in certain inevitable circumstances. Although tigecycline is not officially licensed for the treatment of UTIs, it has demonstrated substantial activity in this case and a few other cases described in the discussion. Given the limited number of available alternatives for effective UTI treatment, additional randomized controlled trials are needed to determine the role of tigecycline in the treatment of complicated urinary tract infections caused by MDR pathogens.

## Data Availability

The original contributions presented in the study are included in the article/Supplementary Material, further inquiries can be directed to the corresponding author.
